# External low energy electromagnetic fields affect heart dynamics: surrogate for system synchronization, chaos control and cancer patient’s health

**DOI:** 10.3389/fnetp.2024.1525135

**Published:** 2025-01-03

**Authors:** Frederico P. Costa, Jack Tuszynski, Antonio F. Iemma, Willian A. Trevizan, Bertram Wiedenmann, Eckehard Schöll

**Affiliations:** ^1^ Oncology Department, Hospital Sírio Libanês, São Paulo, Brazil; ^2^ Dipartimento di Ingegneria Meccanica e Aerospaziale, Politecnico di Torino, Turin, Italy; ^3^ Mathematical and Statistics, Autem Therapeutics, Hanover, NH, United States; ^4^ Physics and Mathematical Modeling, Autem Therapeutics, Hanover, NH, United States; ^5^ Department of Hepatology and Gastroenterology, Charité - Universitätsmedizin Berlin, Berlin, Germany; ^6^ Institut für Theoretische Physik, Technische Universität Berlin, Berlin, Germany

**Keywords:** non-thermal electromagnetic fields, radiofrequency, cancer treatment, cancer cells, oscillations, resonance, synchronization, chaos control

## Abstract

All cells in the human body, including cancer cells, possess specific electrical properties crucial for their functions. These properties are notably different between normal and cancerous cells. Cancer cells are characterized by autonomous oscillations and damped electromagnetic field (EMF) activation. Cancer reduces physiological variability, implying a systemic disconnection that desynchronizes bodily systems and their inherent random processes. The dynamics of heart rate, in this context, could reflect global physiological network instability in the sense of entrainment. Using a medical device that employs an active closed-loop system, such as administering specifically modulated EMF frequencies at targeted intervals and at low energies, we can evaluate the periodic oscillations of the heart. This procedure serves as a closed-loop control mechanism leading to a temporary alteration in plasma membrane ionic flow and the heart’s periodic oscillation dynamics. The understanding of this phenomenon is supported by computer simulations of a mathematical model, which are validated by experimental data. Heart dynamics can be quantified using difference logistic equations, and it correlates with improved overall survival rates in cancer patients.

## Introduction

The human organism functions as an integrated network of organs and systems (Haken, 1977; Haken, 2006; Haken and Portugali, 2016). This hierarchical functional organization of life processes is well described within the formalism of systems biology (Salvador, 2008). Organs, cells, and biomolecules are interacting across different levels to create a dynamic physiological network (Ivanov 2021; Haken, 1977). This system showcases collective behavior emerging from nonlinear and adaptive interactions involving biophysical and biochemical control, along with communication between cells and organs (Schöll, 2022). The human body exhibits a variety of rhythms and oscillations, such as circadian rhythms, cell cycles, and hormone secretion. These oscillators interact within and across tissues and cells, producing a spectrum of behaviors ranging from synchronization to chaos, which are crucial for biological adaptability and evolution. These dynamics, including synchronization phenomena in networks of coupled nonlinear oscillators, are key to understanding biological systems across scales. Such patterns, ranging from cluster synchronization to chimera states with both coherent and incoherent domains, illustrate the universal role of synchronization in both natural and technological contexts (Pikovsky et al., 2001; Rosenblum and Pikovsky, 2004; Boccaletti et al., 2018; Heltberg et al., 2021; Schöll, 2021; Sawicki et al., 2022a; Sawicki et al., 2022b; Berner et al., 2022). Thus, the frequency of oscillation (intrinsic frequency) is the crucial component for understanding the patterns of synchronization, resonance, and chaos. In complex biological systems, endogenous and external electromagnetic fields (EMF) operate as the “fast” primary messenger for physiological and pathological network behavior and control (Costa et al., 2024). Electromagnetic oscillations and synchronization of biomolecules triggered by internal and external pulses are the physical basis of the cellular electromagnetic field (Sun et al., 2022).

Cancer cell behavior has been proposed to be characterized by damped EMF activities (Pokorný et al., 2020). Damped system dynamics in biological systems are influenced by the inherent randomness of biochemical reactions, which disrupt phase coherence and reduce oscillation amplitude. In cancer patients, this leads to an energy deficit in the system, further dampening oscillations and increasing entropy, or disorder. Damping causes reduction in physiological variability that indicates systemic isolation by decoupling systems and their stochastic processes (Pincus, 1994). Physiological system coupling is often measured by variability, especially with heart rate variability serving as a common indicator of physiological variability and overall system stability. Heart rate variability measures the differences between consecutive heartbeats, known as R–R intervals (RRI), over time (Force et al., 1996; D'Angelo et al., 2023; Tiwari et al., 2021).

From the perspective of non-linear dynamics, the human cardiovascular system has a self-oscillatory character at the micro (cellular properties) and macro level. In particular, the heart rate can be synchronized such that the effect of phase locking is observed under the effect of weak external forcing (Anishchenko et al., 2000). The normal sequence and synchronous contraction of heart myocytes (e.g., heart dynamics) results from spontaneous and coordinated rapid flow of ions through ion channels located in the plasma membrane, producing a sequence of action potentials (Grant, 2009). The heart dynamics can be represented by a Van der Pol oscillator (VPO) that is extensively used to model the nonlinear behavior of heartbeats. It is a self-sustained nonlinear dissipative oscillator that exhibits chaotic switching between two types of regular motion, namely, periodic and quasiperiodic oscillations in the principal resonance region under exposure to EMF (Kadji et al., 2007; Van der Pol and Van der Mark, 1927). The driven VPO serves as a paradigmatic model for chaos in low-dimensional systems. When subjected to external forcing (e.g., Lorentz force), VPO can show not only limit cycles (asymptotically stable periodic orbits), but more complex dynamical behavior like strange attractors. Strange attractors, key to nonlinear dynamics and chaotic systems, represent an asymptotic chaotic state with fractal, i.e., non-integer dimension. This results in a system highly sensitive to small changes in initial conditions, leading to practically unpredictable temporal behavior. Cancer cells, in autoregulated damped systems, may be conceived as strange attractors that could interfere directly with the heart dynamics described by a VPO (Uthamacumaran, 2020). This interference leads to phase transition scenarios that determine the system’s initial dynamics prior to EMF exposure (Davies et al., 2011).

A predetermined active closed-loop control system is a type of feedback control system designed to automatically correct any deviations which includes perturbations with feedback dynamics (Gad-el-Hak, 2000). It is more systematically designable, and adaptable to handle noise and uncertainties, making it superior in flexibility and robustness compared to constantly active open-loop control. Closed-loop control uniquely allows for the examination and stabilization of otherwise inaccessible unstable states, offering significant practical and theoretical benefits (Macau and Grebogi, 2007; Schöll et al., 2016; Schöll, 2024). Moreover, it supports the recent advances in controlling low-dimensional chaos in nonlinear systems and its extension to spatiotemporal dynamics. In this study we use a medical device-based methodology to explore the impact of external EMF upon the heart dynamics in cancer patients to assess if heart dynamics could serve as a surrogate for system synchronization, chaos control and ultimate the patient’s initial health status.

## Methods

### Study population

We conducted a retrospective analysis of 22 patients submitted to identical exposure procedure at their first exposure to EMF. This corresponded to a subset of 66 adult patients enrolled in the feasibility trial reported by Capareli et al. (2023). Patients were aged 18 or older with advanced, unresectable, or metastatic hepatocellular carcinoma (HCC), confirmed histologically or clinically per American Association for the Study of Liver Diseases guidelines. They were classified as Child–Pugh A or B cirrhosis, Barcelona Clinic Liver Cancer stage B or C, and an Eastern Cooperative Oncology Group performance status of 0–2. There were no restrictions based on disease progression, hematological or organ function, or previous or current therapies. Hospital Sírio Libanês (São Paulo, Brazil) was the sole study site for the feasibility study where all participants provided written informed consent before enrollment. The trial adhered to the Declaration of Helsinki, with protocol approval by the hospital’s institutional review board, ethics committee, and the Comissão Nacional de Ética em Pesquisa. Following full enrollment, the ethics committee approved a compassionate access program under the same protocol but with a revised consent form. The trial was registered at ClinicalTrials.gov (NCT01686412) prior to starting enrollment. More information about the study population can be found elsewhere (Capareli et al., 2023).

### EMF exposure system

All study participants were exposed to low-energy radio frequency EMF using the AutEMdev prototype (Autem Therapeutics NH United States), a high-precision radio frequency emitter device that controls systemic exposure of a dual signal at a carrier frequency of 27.12 MHz and modulates amplitudes from 10 Hz to 20 kHz (Tuszynski and Costa, 2022). Hemodynamic responses were monitored non-invasively and continuously using a high precision beat-to-beat recording device (Task Force Monitor or CNAP500; CNSystems, Graz, Austria), which was synchronized with the AutEMdev in the millisecond (ms). Patients underwent exposure to a pre-programmed fixed range and sequence of modulation frequencies via the AutEMdev during the discovery phase in part 2 as described by Capareli et al. (2023). Thus, each patient underwent hemodynamic monitoring for 15 min immediately prior to EMF exposure, which consisted of a 15-minute session where they were exposed to a pre-programmed fixed sequence of modulation frequencies delivered by the AutEMdev. The carrier wave amplitude-modulated in sinusoidal form with frequencies ranging from 10 to 100Hz, where each frequency increment of 1Hz was exposed sequentially for 10 s from the lowest to the highest frequency.

### Processing hemodynamic data

Digital data were saved and supported real-time cloud computing that represented a closed-loop control system. The EMF generator emitted a fixed sequence of modulation frequencies, each lasting 10 s over a 15-minute session, simultaneously with hemodynamic monitoring that collected metrics every 10 milliseconds. Every participant’s 15-minute tachogram was automatically processed: the autoregressive method was used for detecting outliers and the electro-pressure vectogram gradient phase time plots were used to study 10-second consecutive periodic orbits as described elsewhere (Capareli et al., 2023; García et al., 2023). This analysis allows the identification of instances of outlier heartbeats with significantly longer or shorter durations than expected. Next, the device produces a patient-specific series of modulation frequencies based on computing the feedback responses collected during the exposure to the fixed series of modulation frequencies. Finally, data recorded from 22 cancer patients, selected from their initial exposure to EMF were used in a retrospective correlation with survival. Full description of the exposure procedures can be found elsewhere (Capareli et al., 2023).

### Model of isolated heart

There is a significant link between the autonomic nervous system and cardiovascular dynamics resulting from sympathetic activity or vagal activity observed in patients with chronic disease, including cancer. However, in order to study potential direct effects of EMF (e.g., external force on the cardiac myocytes), we developed a computational heart model (e.g., isolated heart) focused on the action potential of the cardiomyocytes. The simulation of action potential in cardiomyocytes considers that the membrane is excited beyond a certain threshold, activating ion channels. This leads to ion currents flowing in or out, changing the cardiomyocytes’ potential and triggering an action potential. After this, the membrane potential resets to its resting state, ready for another excitatory input. During an action potential, the cardiomyocyte enters a refractory phase where it is temporarily unresponsive to external disturbances. The rate at which these cells depolarize varies, affecting how quickly they generate action potential. Thus, the action potential as a function of the time, where the shape of the action potential remains constant while the frequency may be changed in a wide range.

### Time-continuous computer modeling

**FIGURE 1:**
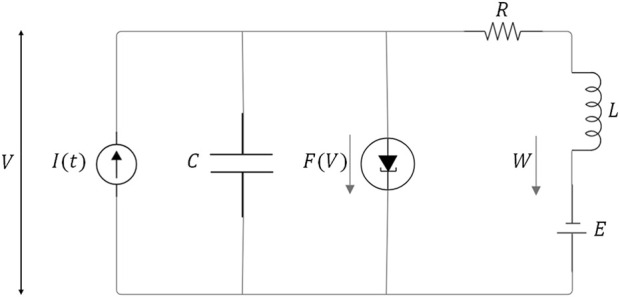
Schematics of the electronic analogue for the FitzHugh-Nagumo model.

A computational heart model simulated the EMF signal coupling with the cardiomyocytes and their action potentials following Hodgkin-Huxley, FitzHugh-Nagumo, and modified VPO equations. The oscillations, variable excitability, refractoriness, and asymmetric action potentials with distinct depolarization and repolarization rates were validated to reproduce experimental data (García et al., 2023; Berenfeld and Abboud, 1996; Kumai, 2017). In the following we focus on the FitzHugh-Nagumo model, which simplifies the complex dynamics of ionic voltage-gated channels described by the Hodgkin-Huxley equations, by means of an electronic analogue that captures two basic processes (Figure 1). 1) A current $F\left(V\right)=\frac{V^{3}}{3}-V$ that depends nonlinearly on the membrane potential V (activator), and responds in phase with it, representing the relatively fast opening of gated channels that allow a current influx in the cell, and 2) a delayed and persistent current W (inhibitor) that restores equilibrium in a longer time-scale, representing the slow response of channels closing (especially the potassium channels), which is modeled through an effective in-parallel inductor L and resistance R. A battery E adjusts the value of the resting potential of the membrane.

$$\epsilon \frac{dV}{d\tau }=I\left(\tau \right)-F\left(V\right)-W$$

$$\frac{dW}{d\tau }=V+E-RW$$ (1)

where τ is a rescaled time variable defined as τ=t/L . This definition introduces the parameter ϵ=C/L in the dynamical equation for the potential, which expresses the ratio between the capacitive and inductive relaxation times, making the time scale separation between the two gating processes explicit.

The FitzHugh-Nagumo model allows the input of an external current into the system, which in our case is an ionic current in phase to the local electric field delivered in the proximities of the membrane, therefore having the form:

$$I\left(\tau \right)=A\left(\tau \right)\cos\left(\omega_{\;c}\tau \right)$$ (2)

where $\omega_{\;c}$ is the carrier wave angular frequency, and $A\left(\tau \right)$ is the modulated envelope that evolves at a much slower pace.

## Results

### Time-continuous computer modeling

**FIGURE 2:**
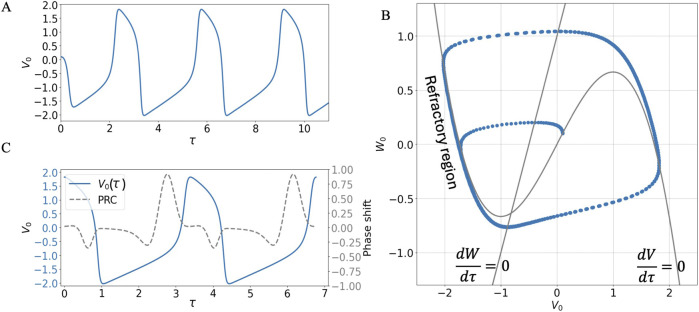
Evolution towards the limit cycle of an unforced solution of the FHN model with parameters ϵ=0.1 , E=0.5 and R=0.5 **(A)**. Time series $V_{0}\left(\tau \right)$ representing the evolution of the action potential **(B)**. Representation in the $\left(V_{0}\left(\tau \right),W_{0}\left(\tau \right)\right)$ phase space highlighting the refractory region of the limit cycle **(C)**. Phase Response Curve (PRC) superimposed to the limit cycle solution of the model. Near the refractory region, short external stimuli have little to no effect on the undriven trajectory. Before the ascending and descending parts of the action potential, an external stimulus can either advance or retard the trajectory, respectively, depending on the sign of the PRC.

In the absence of an external oscillating current I = 0 and with suitable choice of parameters, the solution of Equation 1 for the action potential evolves as an oscillating function $V_{\;0}\left(t\right)$ in a characteristic time scale of the order of a second, reaching a limit cycle, as shown in Figures 2A, B (in the simulations for this section the parameters were set to ϵ=0.1 ; E=0.5 ; R=0.5 ).

This trajectory is a combination of a slow and a fast dynamic part, as seen in the time series of Figure 2A and in the phase portrait of Figure 2B, which arises as a combination of a slow motion (order of ϵ ) close to the two branches of the $W=V-V^{3}/3$ nullcline with negative slope, and a fast motion when the trajectory of $\left(V_{0}\left(\tau \right),W_{0}\left(\tau \right)\right)$ detaches from the $W=-F\left(V\right)$ nullcline (Schöll et al., 2009; Omelchenko et al., 2013). A refractory region then emerges near the left branch of the nullcline, highlighted in Figure 2B, during which the system is expected to be more robust to external stimuli. Outside the slow refractory part of the trajectory and outside the slow part of the excitation pulse (right branch of the nullcline), the system becomes vulnerable to an external EMF oscillating stimulus.

**FIGURE 3:**
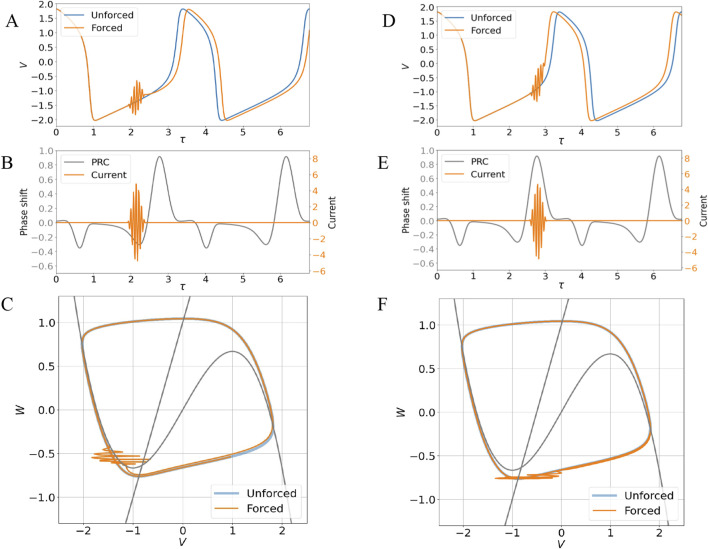
**(A)**. Comparison between the limit cycle solution for the undriven system (unforced) and the delayed trajectory forced by a short external stimulus of the form of Equations 2, 3 **(B)**. Short current stimulus applied during a negative portion of the PRC. The parameters for the current are $A_{0}=5$ , $\omega_{\mathrm{mod}}=12.6$ and $\omega_{c}=88.0$ **(C)**. Representation of the trajectories in the phase space **(D)**. Comparison between the limit cycle solution for the undriven system (unforced) and the advanced trajectory forced by a short external stimulus of the form of Equations 2, 3 **(E)**. Short current stimulus applied during positive portion of the PRC. The parameters for the current are $A_{0}=5$ , $\omega_{\mathrm{mod}}=12.6$ and $\omega_{c}=88.0$ **(F)**. Representation of the trajectories in the phase space.

The robustness/vulnerability to external stimuli can be inspected by analyzing the system’s Phase Response Curve (PRC), which quantifies the phase shifts induced in the trajectory due to very short perturbations ( δ -function like stimuli), and that can be computed by solving for the unstable periodic limit cycle of the adjoint system (Schultheiss et al., 2012; Winkler et al., 2022). The calculated Phase Response Curve in Figure 2C shows that a weak stimulus will have little effect in the action potential when applied in the refractory region or during the slow part of the excitation pulse. When the trajectory detaches from the slow regions near the two negative-slope branches of the nullcline, a weak stimulus may either retard or advance the action potential depending on whether the sign of the Phase Response Curve is negative or positive, respectively. Figures 3A–C and 3D-F show these effects (delayed and accelerated action potentials, respectively) when an external current like the one in Equation 2 is applied for a short time of one cycle of the envelope function, which takes the form of:

$$A\left(\tau \right)=\frac{A_{0}}{2}\left(1+\cos\;\left(\omega_{\mathrm{mod}}\tau \right)\right)$$ (3)

When subject to an external current of the form of Equation 2, the forced solutions $\left(V\left(\tau \right),W\left(\tau \right)\right)$ evolve on a fast time scale imposed by the carrier frequency and a slow time scale which governs average deviations from the free system (Figures 3A–C and 3D-F). The effective pull felt by the system in the slower time scale in the presence of the EMF stimulus can be modeled by substituting the external current by an effective current, obtained by averaging out the fast time scale in the original dynamical equations (derivation in the following section):

$$I_{eff}\left(\tau \right)=-KA^{2}\left(\tau \right)V\left(\tau \right)$$ (4)

K being a coupling parameter. This introduces a closed-loop control since the driving term depends upon the system variable V . The effective driving current evolves at a time scale closer to that of the system and is similar to a train of pulses bursting at the envelope frequency.

**FIGURE 4:**
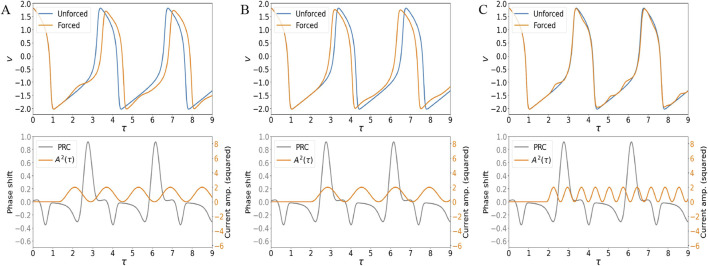
Comparison between the limit cycle solution for the undriven system (unforced) and the trajectory forced by a continuous external stimulus represented by an effective current (Equation 4), with envelope evolving as Equation 3. Parameters K=0.03 and $A_{0}=1$ . **(A)** $\omega_{\mathrm{mod}}=3.9$ and starting point of the envelope were chosen to suppress the current train in most of the positive regions of the PRC, leading to a delayed action potential. **(B)** $\omega_{\mathrm{mod}}=3.7$ and starting point of the envelope were chosen to suppress the current train in most of the negative regions of the PRC, leading to an advanced action potential. **(C)** $\omega_{\mathrm{mod}}=9.0$ and starting point of the envelope were chosen as to leave the action potential unaffected.

It has been shown that a train of pulses at appropriate frequencies and phases is able to induce time-changes in the orbit of modified Van der Pol systems (Zebrowski et al., 2007). In fact, Figures 4A–C show the impact of the effective current when the envelope of Equation 3 is applied continuously. When the frequency and starting point of the envelope train are chosen such that the external effective current is nearly zero for most of the positive regions of the Phase Response Curve, the action potential is slowed down (Figure 4A). Note that the sign in Equation 4 makes the current impulse positive whenever V<0 . Different choices in parametrization can also speed up the action potential (when the effective current is suppressed in the negative regions of the PRC (Figure 4B), or do not affect it (Figure 4C).

Therefore, the proposed EMF coupling can model changes in heartbeat intervals observed experimentally (Figure 8), based on ion flow dynamics. Furthermore, if a significant difference in the model parameters is expected (e.g., normal vs. cancer cells bioelectric properties), we can likely consider radical behavioral shifts in ion flow dynamics across the membrane.

### Derivation of the effective current

When the EMF signal is delivered, the forced solution of Equation 1 can be expressed in terms of the deviations $\left(V_{1},W_{1}\right)$ from the undriven free system, that is, $V\left(\tau \right)=V_{0}\left(\tau \right)+V_{1}\left(\tau \right),W\left(\tau \right)=W_{0}\left(\tau \right)+W_{1}\left(\tau \right)$ with the perturbation terms satisfying:

$$\epsilon \frac{dV_{1}}{d\tau }=I\left(\tau \right)-G\left(V_{0},V_{1}\right)-W_{1}$$

$$\frac{dW_{1}}{d\tau }=V_{1}-RW_{1}$$

$$G\left(V_{0},V_{1}\right)=F\left(V_{0}+V_{1}\right)-F\left(V_{0}\right)$$ (5)

As shown in Figures 3A, D, because of the difference between the carrier wave frequency $\omega_{c}$ and all the other time scales of the system, the solutions of Equation 5 are expected to evolve at two different time scales, which we will call $\tau_{F}=\tau$ (fast) and $\tau_{S}=\frac{1}{\omega_{c}}\tau$ (slow). Treating them as independent variables in a dual time approach, one can write $V_{1}\left(\tau \right)={V_{1}}^{\left(0\right)}\left(\tau_{F},\tau_{S}\right)+\frac{1}{\omega_{c}}{V_{1}}^{\left(1\right)}\left(\tau_{F},\tau_{S}\right)+...$ and $W_{1}\left(\tau \right)={W_{1}}^{\left(0\right)}\left(\tau_{F},\tau_{S}\right)+\frac{1}{\omega_{c}}{W_{1}}^{\left(1\right)}\left(\tau_{F},\tau_{S}\right)+...$ ,which has a solution in the lowest order in $\frac{1}{\omega_{c}}$ of the form:

$${V_{1}}^{\left(0\right)}\left(\tau_{F},\tau_{S}\right)=\frac{A}{{\epsilon \omega }_{c}}\sin\left(\omega_{c}\tau_{F}+\delta \right)+a\left(\tau_{S}\right)$$

$${W_{1}}^{\left(0\right)}\left(\tau_{F},\tau_{S}\right)=b\left(\tau_{S}\right)e^{-R\;\tau_{F}}+\frac{1}{R}a\left(\tau_{S}\right)$$

$$a\left(0\right)=-R\;b\left(0\right)=-\frac{A}{\epsilon \omega_{c}}\sin\;\delta$$ (6)

Here, the initial time was chosen such that the perturbations were absent ( $V_{1}=0$ , $W_{1}=0$ ), when the EMF current was delivered to the system at an arbitrary phase δ . Equation 6 show that in the presence of the external EMF current, the potential responds as a fast wave that oscillates around a slower changing function $a\left(\tau_{S}\right)$ , as in Figures 3A, D.

Moving up one order in the dual time approach, it can be shown that:

$$\frac{1}{{\epsilon \omega }_{c}}\frac{dA}{d\tau_{S}}\sin\left(\omega_{c}\tau_{F}+\delta \right)+\frac{da}{d\tau_{S}}=-\frac{\omega_{c}}{\epsilon }\left(G|\left(V_{0},{V_{1}}^{\left(0\right)}\right)|+|{W_{1}}^{\left(0\right)}\right)$$ (7)

Averaging over the fast variable (one cycle of $\tau_{F}$ ) and over all the possible initial conditions $\left(0\leq \delta <2\pi \right)$ , Equation 7 yields (in the original time variable τ and for very short times):

$${\epsilon \frac{da}{d\tau }}_{\tau \to 0}=-{\left(\frac{1}{{\epsilon \omega }_{c}}\right)}^{2}A^{2}\left(0\right)\;V_{0}\left(0\right)$$ (8)

Therefore, the fast-varying current exerts an effective pull on the slow time evolution of the perturbation that is proportional to the potential (and opposite in sign with it), and proportional to the square of the envelope amplitude. Equation 8 motivates the substitution of the exact current in the original system (Equation 1) by its effective slower evolving counterpart (Equation 4).

### Time-discrete mathematical modeling

Considering a train of pulses at appropriate frequencies and phases that interferes with the heart dynamics causing changes in heart rate variability, by time-domain, frequency domains and nonlinear analysis, we examine how the heart’s qualitative behavior changes with variations in action potential trajectories.

**FIGURE 5:**
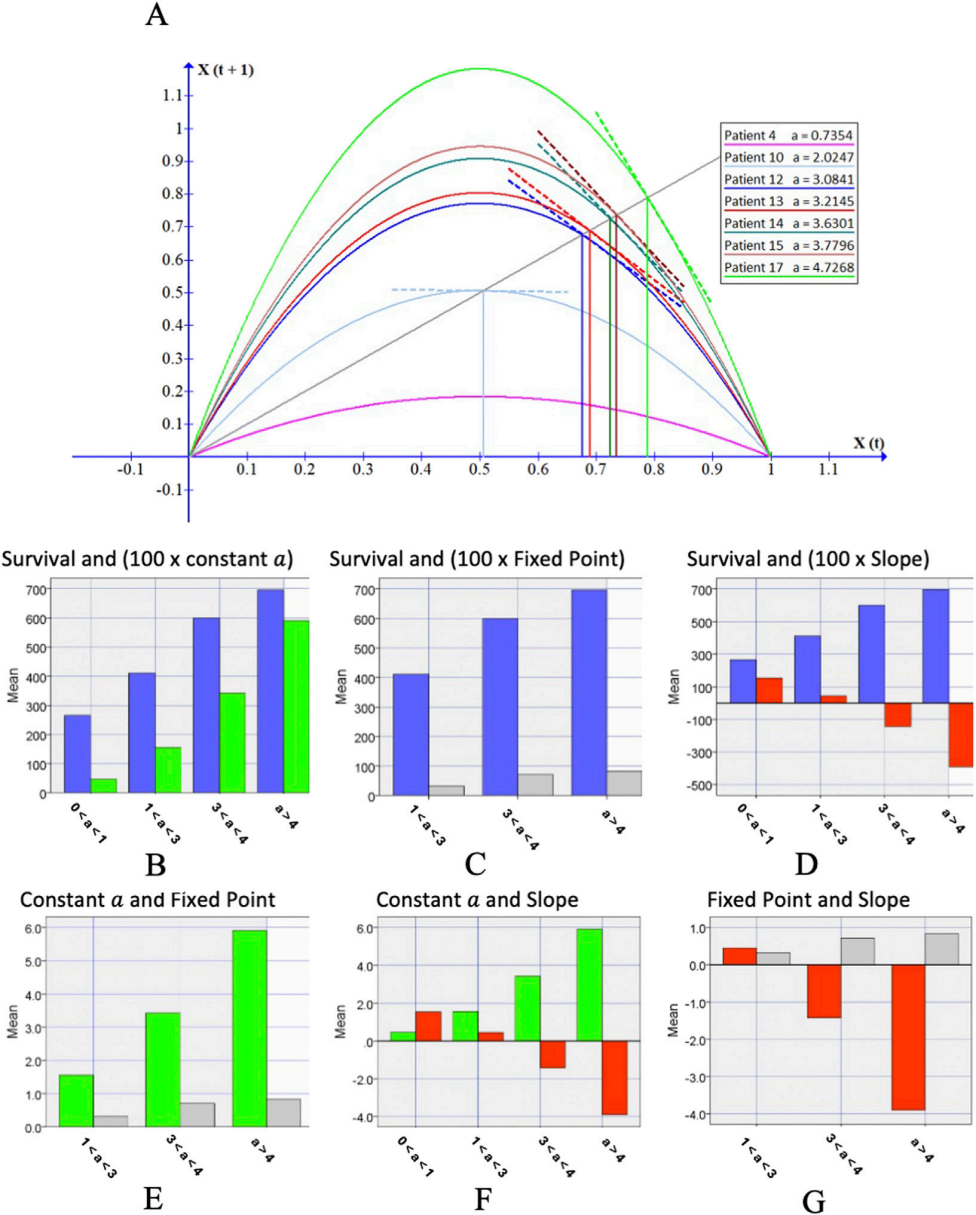
**(A)**. displays Difference Equation curves x_t+1_ = ax_t_ (1−x_t_) for seven cancer patients, each characterized by a specific growth rate constant a . The intersection points of these curves with the line f(x) = x identify fixed points (orthogonal projections on the X(t) axis) and their slopes indicate the stability of these points (dashed lines). Figures **(B–D)** illustrate strong correlation trends: a positive correlation between survival rates and the growth rate constant a and fixed points, and a negative correlation between the slope and survival rates. Figures **(E–G)** further highlight these trends, showing strong positive correlations between the fixed points and the constant a , and strong negative correlations between the slope, the constant a , and the fixed points. See Supplementary Table 1S and Supplementary Figure 1S for more information. Blue bars corresponds to mean survival. Green bars corresponds to mean constant a values.

A difference equation model discretizes events in time by expressing each term as a function of its immediately preceding term, enabling step-by-step computation of the sequence (Buchner and Jan Żebrowski, 2001; Buchner and Żebrowski, 2000; May, 1976). We use the logistic function for the interval x of two successive spikes:

$$F\left(x\right)=\text{ax }\left(1\;–\;x\right)$$

which gives the logistic difference equation, defined for 0<x<4, and has the form:

$$x_{t+1}={\text{ax}}_{t}\;\left(1\;‐\;x_{t}\right)$$

where non-trivial behavior occurs only if 1<a<4 and in this case, the fixed point is $x^{*}=1-\frac{1}{a}$ . as illustrated in Figure 5.

Four strata were considered: If 0 < a < 1 then there is only the trivial solution x = 0, to which all orbits are attracted. If 1 < a < 3 the fixed-point x * is stable and attracts all orbits. If 3 < a < 4 stability is defined by the curve slope, S = 2 - a, at the fixed point and bifurcations and chaos may occur. And if a > 4 then the fixed point tends to infinity (Supplementary Figure 1S, supplement information). Constant a was calculated for each patient, as a function of sample estimates of time to event in seconds, meaning the time for low-frequency signal exposure where an outlier event was observed: median, standard deviation, coefficient of variation and skewness (Supplementary Table 1S, supplement information). It was observed a strong positive correlation between the averages of the strata of a and the corresponding average survival of patients as illustrated in Figure 5.

### Experimental data validation

**FIGURE 6:**
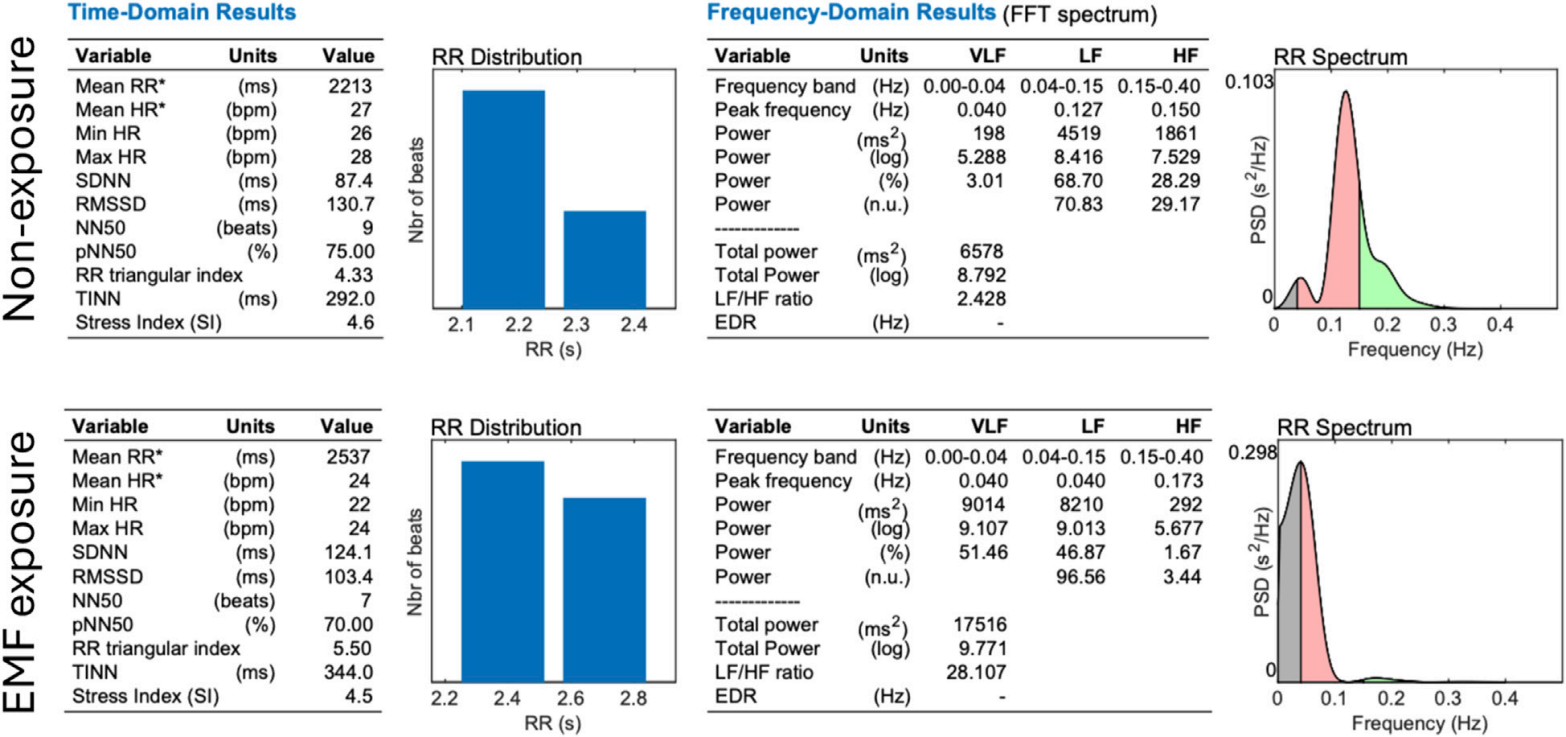
Comparison between non-exposure vs. exposure 15-minute tachograms recorded from a cancer patient. EMF exposure caused a delay in the heart periodic oscillations. In the time-domain, EMF interference resulted in increase in RRI duration, increase in SDNNI, decrease in RMSSD. In the frequency-domain, there is a shift for high VLF power, large increment in total power and LF/HF ratio. Graphic produced by Kubios. Note: RRI: R-R intervals; RMSSD: Square root of the mean squared differences between successive RR intervals; SDNNI: Mean of the standard deviations of RR intervals in 5-min segments; VLF: very low band peak frequencies; LF: low frequency band peak frequencies; HF: high frequency band peak frequencies.

**FIGURE 7:**
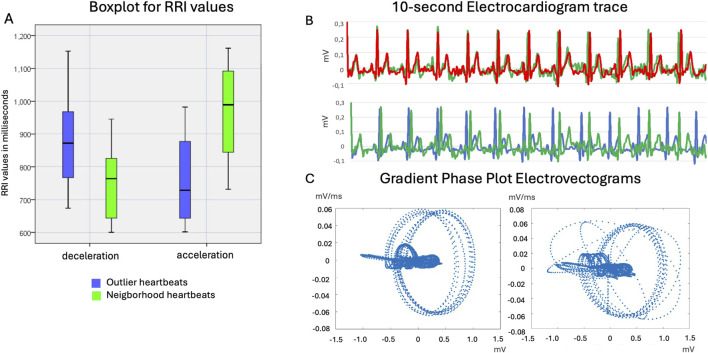
**(A)** The boxplot displays outlier heartbeats categorized into decelerated and accelerated types, compared to the neighboring R-R interval values. The neighboring beats consist of a total of 8–10 heartbeats, divided equally before and after the outlier heartbeat. The comparison of two sequential 10-second tachograms shows **(B)** progressive larger RRI and **(C)** trajectory effect in gradient phase plot vectograms, when no EMF effect is observed, or the deceleration effect is introduced into the time series of heart beats.

**FIGURE 8:**
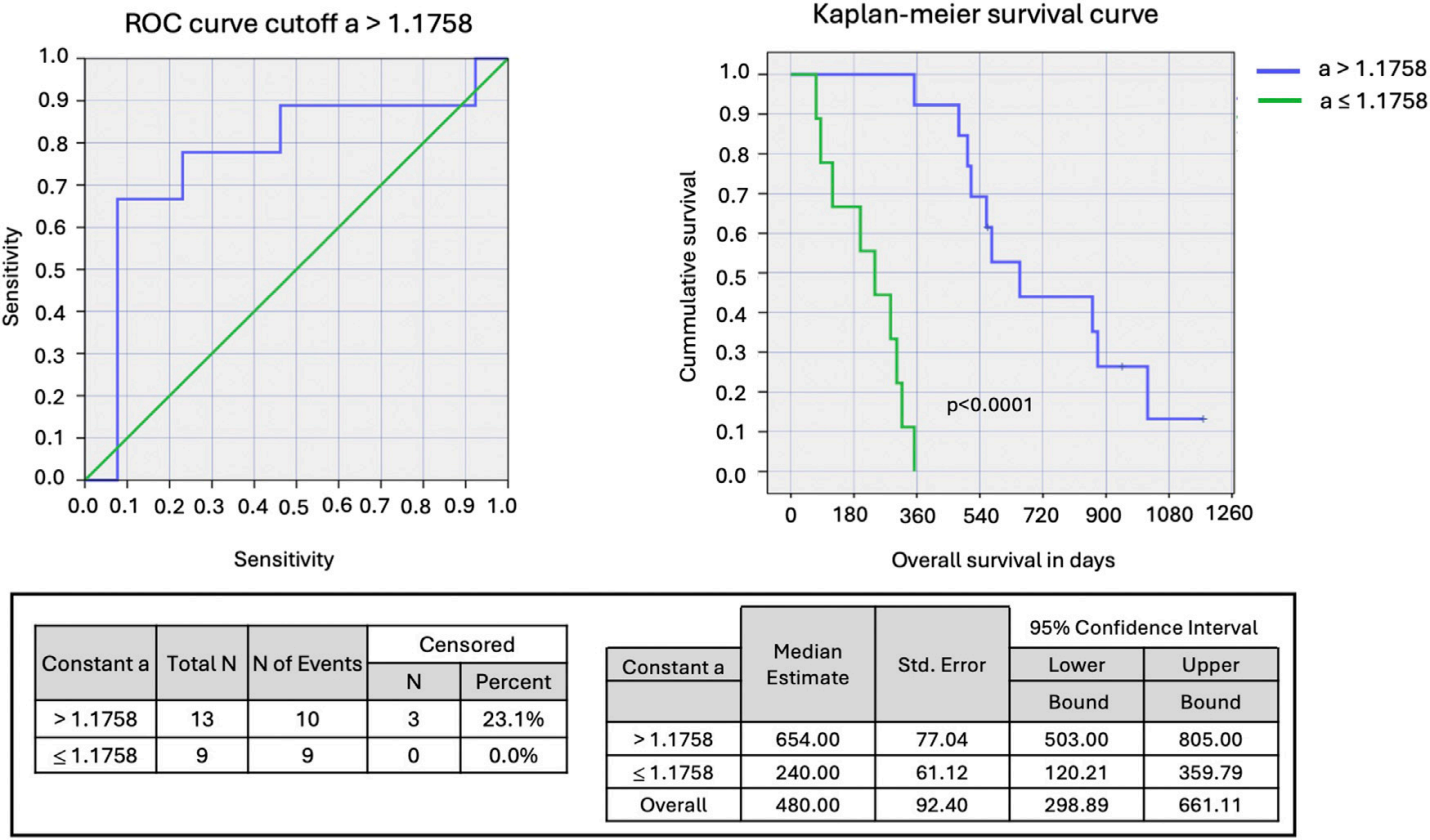
A ROC curve for 22 cancer patients is presented (left). The Kaplan-Meier curve showed significantly better overall survival for cancer patients with constant a > 1.1758.

The RRI time series displayed a nonlinear, quasi-periodic oscillation with an average RRI of 813.257 ± 143.593 milliseconds. We observed a shift in heart rate variability when comparing conditions of non-exposure and exposure in the same patient as illustrated in Figure 6, representing a change in nonlinear dynamics of the heart induced by EMF modulation. Further results are detailed in Capareli et al. (2023). Outlier heartbeats were identified from each participant’s 15-minute tachogram and are summarized in Table 1. All participants exhibited outlier heartbeats, either as isolated incidents or cluster of events (burst), associated with specific frequencies of amplitude modulation exposure. These outlier heartbeats were significantly longer or shorter than neigborhood RRI, as shown in Figure 7. Additionally, there were more instances of decelerated (longer) outlier heartbeats than accelerated (shorter) ones. However, the mean difference in RRI was in the order of +16.57 ± 27.97 milliseconds. A number of heart rate variability metrics for time domain, frequency domain and nonlinear analysis were studied as potential predictors for <360-day survival from the first exposure, using data from patients with advanced hepatocellular carcinoma restricted to the first exposure for each patient p=1,2,....22. However, all of the heart rate variability metrics showed a low predictive accuracy determined by Receiver-Operating Characteristic (ROC) curve. On the other hand, when a difference logistic equation was used, there was a significant improvement in accuracy (Table 2). A ROC curve was constructed using the constant a with a cutoff value set at 1.758. Patients with a value greater than 1.758 had a median overall survival of 21.5 months (CI 95%: 16.5 < > 26.4), compared to 7.9 months for those with a value of 1.758 or less (CI 95%: 3.9 < > 11.8) (Figure 8). This difference in survival rates was statistically significant, as indicated by a Log Rank test (p < 0.0001). The constant a demonstrated very effective predictive power for longer survival times, particularly those related to specificity (0.9231, CI 95%: 0.7782 < > 1.0000), positive predictive value (0.8571), negative predictive value (0.8000) and likelihood ratio for positive tests (8.667) (Figure 8). We observed significant correlation between sample entropy and constant a (2-tailed Person’s correlation, p = 0.035) (Supplementary Table 2S, supplement information).

**TABLE 1:** Description of outlier heart beats identified by automated process in the 15-minute tachograms recorded from each participant during their first EMF exposure.

Patient’s initial	Outlier heart beats				Neighborhood beats		Outcome
	Single	Burst	Mean	Std. Dev	Mean	Std dev	
HMC	7	4	684.91	22.67	731.53	20.43	Accelerated
NJP	13	2	889.96	207.99	800.42	31.02	Decelerated
PJF	28	4	1152.69	72.77	783.56	32.43	Decelerated
LGM	18	8	673.83	61.98	601.22	146.07	Decelerated
LS	24	24	789.70	53.73	686.07	22.57	Decelerated
VML	24	4	988.12	53.73	945.52	41.31	Decelerated
APG	14	1	772.16	56.05	956.88	62.57	Accelerated
MA	27	18	854.37	61.20	850.62	11.64	Decelerated
JAS	19	5	601.79	34.60	1161.63	34.78	Accelerated
CBC	35	8	744.21	30.92	600.40	12.05	Decelerated
GF	35	2	948.23	80.56	743.88	19.00	Decelerated
JBC	27	9	982.50	81.33	1021.82	48.06	Accelerated
General	271	89	840.20	68.13	823.63	40.16	Decelerated

**TABLE 2:** Sensitivity and specificity by ROC curve for prediction of survival longer than 360 days from initial EMF exposure procedure in 22 patients with advanced HCC.

Time and nonlinear domains	Cutoff	Sensitivity	Especificity
S_Entropy	≥1.2549	0.7000	0.6667
Higuchi	≥1.2549	0.7000	0.6667
MFDFA	≤1.2335	0.9000	0.4167
RMSSD	≤10.5739	0.3000	0.8333

Frequency domains	Cutoff	Sensitivity	Especificity
VLF	≤227.0441	0.9000	0.5000
LF nu	≤63.4010	0.6000	0.5833
HF nu	≥30.4039	0.6000	0.5833
LF/HF	≤1.7323	0.6000	0.5833
Total power	≤213.6301	0.5000	0.6667
**Constant** a	**≤1.1758**	**0.6667**	**0.9231**

Note: S_Entropy: sample entropy; Higuchi, Higuchi fractal dimension; MFDFA, modified detrended fluctuation analysis, RMSSD, root mean square of successive differences between normal heartbeats; VLF, very low frequency spectrum; LF, nu: indexed low frequency spectrum, HF, nu: indexed high frequency spectrum; LF/HF, low frequency high frequency ratio.

## Discussion

This retrospective study with 22 cancer patients is the first to demonstrate a closed-loop control method to exam the heart dynamics under the exposure to an external low energy EMF signal at appropriate frequencies and phases acting upon the limit cycle attractor of the oscillating heart.

This noninvasive, safe and simple method provides for the first time a measurement of the deterministic interference with the ionic flow dynamics of the plasma membrane observed in excitable cells (e.g., cardiomyocytes) with a large potential of application in medicine (Mattsson et al., 2009). Moreover, the determination of heart dynamics in this setting may serve as a novel strategy to access not only cancer patients’ health status but a novel cancer treatment approach. Therefore, this novel technology could open a new treatment area combining diagnostics with therapy using systemic EMF signals (e.g., theranostics).

The heart generates by far the strongest EMF in the body, surpassing the brain’s output in both electric and magnetic strength. As the predominant source of EMF oscillations, the heart produces nonlinear interactions affecting the entire complex system’s behavior of a human being (McCraty, 2016). The heart may act as a predominant node in the body’s physiological EMF network (Schubert, 1993; Becker and Selden, 1985). The heart’s dominant EMF oscillations exert influence over practically all EMF (also called bioelectromagnetic fields) produced by the human body through its biological processes because of the electrical activities of the cells, tissues, and organs (Young et al., 2021; Abdul Kadir et al., 2018; Kaestner et al., 2018). By introducing resilient limit cycle attractors at appropriate frequency and phase determined by feedback, one can leverage the sensitivity of chaotic systems to initial conditions and provide control of chaos through feedback control. These interventions are aimed to modify the system’s energy landscape, potentially stabilizing chaotic fluctuations, and steering the system towards predictable and regular behavior. This method uses minimal energy, and it is yet crucial for enhancing control in systems where chaos is a disruptive force, making it applicable to the entire physiological system (e.g., human body). This study presents a straightforward control strategy for managing chaotic oscillations in systems such as cardiac rhythms by stabilizing a previously unstable periodic behavior inherent in the system’s natural dynamics. Our approach uses a series of pulse trains that introduce delays in the heart dynamics, potentially leading to the natural stabilization of the periodic orbit, reducing the system’s chaotic fluctuations with minimal energy loss (Pyragas, 1992; Pyragas and Tamaševičius, 1993; Pyragas, 2006; Schöll and Schuster, 2008; Purewal et al., 2014).

Heart dynamics alterations induced by EMF occur significantly more frequently in cancer patients than in healthy volunteers as observed by Capareli et al. (p < 0.0001) (Capareli et al., 2023). Low-energy EMF appear insufficient to disrupt heart dynamics in healthy volunteers, based on our experimental observation. This suggests that internal oscillators in healthy physiological states, governed by stable dynamics with feedback and flexibility, are robust and adaptable to external interference. However, as in disease, damped systems exhibit instabilities that can drive physiological networks toward phase transitions, making them more susceptible to smooth subtle external disturbances (Pokorný et al., 2020). Furthermore, the initial conditions of a system significantly influence its long-term behavior, particularly in damped systems. This importance arises from the nonlinear dynamics of these systems, where even minor differences in initial states can result in dramatically divergent outcomes, which is referred to as sensitivity to initial conditions. Consequently, by examining the heart dynamics as response to the first exposure to EMF, we can gain insights into the individual health status as reflected in the dynamics of the system’s responses. Besides, big data containing frequency-time information combined with clinical outcomes represent a powerful and unique data source. Patients exhibiting different dynamics such as bifurcation of periodic oscillations, odd-cycle periodic windows or other dynamics, appear to experience better survival outcomes. Due to the limitations of the existing experiments, the long-term effects at various timescales, such as on circadian rhythms, cannot be determined from the current clinical data. Further research using a larger dataset in prospective clinical trials is required to explore the effects of EMF on survival outcomes and long-lasting effects.

As to the underlying molecular mechanism, the resting membrane potential V_m_ varies across and within cell types. Mature ‘quiescent’ cells, such as neurons and cardiomyocytes, typically maintain a membrane potential V_m_ of about -70 mV, while cancer cells are significantly more depolarized, V_m_ in resting state ranging from −50 to −10 mV or even less (Di Gregorio et al., 2022). Additionally, cancer cells demonstrate heightened electrical activity, with evoked V_m_ ranging from 100 to 400 μV in comparison to excitable cells ranging in mV (Restaino et al., 2023). Unlike normal cells, the membrane potential of cancer cells fluctuates more frequently, with hyperpolarization events occurring 2 ± 0.2 times per cell per 1,000 s and showing about a 3% variation in V_m_ (Quicke et al., 2022; Quicke et al., 2021). Analysis of cellular V_m_ time series indicates both synchronous and asynchronous intercellular crosstalk between cancer cells, with temporal events ranging from 0.01 to 1 Hz (Quicke et al., 2022). Additionally, within the realm of cancer, membrane depolarization may play a crucial role in the development, proliferation and persistence of cancer stem cells, contributing to continuous tumor growth (Yang and Brackenbury, 2013). Given that the systemic electric field generated by the heart is around 50 mV/m, and the external EMF signal delivers an electric field energy output 100 times higher, we hypothesize that a train of pulses at specific frequencies and phases could modulate the membrane potential through ionic flow dynamics of cancer cells, reverting it to a hyperpolarized V_m_ through the activity of voltage-gated channels as observed in the heart (Tsong, 1988; Nawarathna et al., 2005). It is of note here that 5V/m produced by the AutEMdev is below the safety limits of EMF exposure in humans (Mattsson et al., 2009). In fact, the EMF coupling term of Equation 4 favours damping of the action potential trajectory, depending on the bioelectrical parameters of the cell (hence the resting potential), which may drive the action potential towards a polarized state. Furthermore, translational studies support the concept that the effects of EMF under the given conditions cause a calcium flux in cell culture and xenograft models leading to an activation of calcium gated channels (Jimenez et al., 2019; Sharma et al., 2019).

In conclusion, the presented data offer a new theranostic concept by a minimally invasive and rapid method of controlling ionic flow dynamics via the plasma membrane with a large potential of application in medicine. By assessing heart dynamics, this method potentially allows the diagnostic recognition of individual EMF in cancer patients (e.g., patient-specific frequencies) thereby finding new means for the translation of these diagnostic signals into therapy and possibly prognostication by EMF.

## Data Availability

The original contributions presented in the study are included in the article/Supplementary Material, further inquiries can be directed to the corresponding author.
