# Supplementary material for: External low energy electromagnetic fields affect heart dynamics: surrogate for system synchronization, chaos control and cancer patient’s health
Source: Front Netw Physiol. 2025 Jan 3;4:1525135. doi: 10.3389/fnetp.2024.1525135 (PMC11739291; doi:10.3389/fnetp.2024.1525135)
Supplement: Supplementary file 1 [file Table1.docx]

**Supplement information**

Figure 1S. Representation of patient’s dynamic behavior with the application of different logistic equation.

| Constant $a$ | Patient’s dynamics |
| --- | --- |
| ß 1.0000 | Orbits attracted to the trivial solution x = 0 |
| Between 1 and 3 | The fixed point is stable and attracts all trajectories |
| 3.000 | The fixed point becomes unstable. Start of the chaotic region |
| 3.570 | Accumulation point of cycles with periods 2^n |
| 3.678 | The first odd-period cycle appears |
| 3.284 | The period-three cycle appears |
| 4.000 | End of the chaotic region |
| > 4.000 | The fixed point tends towards infinity |

Table 1S. Results from logistic difference equation for 22 cancer patients at their first exposure to EMF.

| Magnitude | Patient’s initial | Patient_ID | OS (Days) | Status | Constant a | Fixed point | Slope |
| --- | --- | --- | --- | --- | --- | --- | --- |
| 0 < a < 1 | LS | 1 | 654 | 1 | 0.0167 | There is no  in the  0 < x < 1  interval | 1.9833 |
|  | MA | 2 | 199 | 1 | 0.2461 |  | 1.7539 |
|  | RS | 3 | 285 | 1 | 0.4481 |  | 1.5519 |
|  | JP | 4 | 119 | 1 | 0.7354 |  | 1.2646 |
|  | MAu | 5 | 72 | 1 | 0.8387 |  | 1.1613 |
| 1 < a < 3 | VR | 6 | 85 | 1 | 1.0601 | 0.0567 | 0.9399 |
|  | PJF | 7 | 303 | 1 | 1.1871 | 0.1576 | 0.8129 |
|  | EB | 8 | 505 | 0 | 1.4203 | 0.2959 | 0.5797 |
|  | JAS | 9 | 861 | 1 | 1.4856 | 0.3269 | 0.5144 |
|  | SS | 10 | 353 | 1 | 2.0247 | 0.5061 | -0.0247 |
|  | VLG | 11 | 359 | 1 | 2.1557 | 0.5361 | -0.1557 |
| 3 < a < 4 | CMR | 12 | 574 | 1 | 3.0841 | 0.6758 | -1.0841 |
|  | NJP | 13 | 946 | 1 | 3.2135 | 0.6888 | -1.2135 |
|  | MS | 14 | 318 | 1 | 3.6301 | 0.7245 | -1.6301 |
|  | HMC | 15 | 559 | 1 | 3.7796 | 0.7354 | -1.7796 |
| a > 4 | MACT | 16 | 480 | 1 | 4.1471 | 0.7589 | -2.1471 |
|  | KSV | 17 | 876 | 1 | 4.7268 | 0.7884 | -2.7268 |
|  | JBC | 18 | 1178 | 0 | 5.5348 | 0.8193 | -3.5348 |
|  | MNO | 19 | 1019 | 1 | 6.5053 | 0.8463 | -4.5053 |
|  | PA | 20 | 515 | 1 | 6.6076 | 0.8487 | -4.6076 |
|  | JCC | 21 | 240 | 1 | 6.6778 | 0.8503 | -4.6778 |
|  | LG | 22 | 562 | 0 | 7.1311 | 0.8598 | -5.1311 |

Note: OS: overall survival; status 0: alive 1: dead; fixed point defined by x* = 1 – 1/a; inclination defined by l = 2 – a.

Table 2S. Pearson Correlation between different HRV metrics and constant $a$ for 22 cancer patients at their first exposure to EMF.

|  | | SEntropy  RRI | Higuchi  RRI | mfdfa  group_RRI | RMSSD  RRI | LF/HF | LFnu | HFnu | Total  power | VLf | IAP |
| --- | --- | --- | --- | --- | --- | --- | --- | --- | --- | --- | --- |
| SEntropy  RRI | Pearson Corr. | 1 | .528 | -.363 | .278 | -.302 | -.247 | .247 | .248 | -.128 | .451 |
|  | Sig. (2-tailed) |  | .012 | .097 | .211 | .172 | .267 | .267 | .265 | .569 | .035 |
| Higuchi  RRI | Pearson Corr. | .528 | 1 | -.912 | .440 | -.613 | -.819 | .819 | .001 | -.360 | .212 |
|  | Sig. (2-tailed) | .012 |  | .000 | .041 | .002 | .000 | .000 | .996 | .100 | .344 |
| MFDFA  group_RRI | Pearson Corr. | -.363 | -.912 | 1 | -.477 | .659 | .907 | -.907 | -.018 | .393 | -.276 |
|  | Sig. (2-tailed) | .097 | .000 |  | .025 | .001 | .000 | .000 | .937 | .070 | .214 |
| RMSSD  RRI | Pearson Corr. | .278 | .440 | -.477 | 1 | -.382 | -.544 | .544 | .787 | .158 | .030 |
|  | Sig. (2-tailed) | .211 | .041 | .025 |  | .079 | .009 | .009 | .000 | .482 | .894 |
| LF/HF | Pearson Corr. | -.302 | -.613 | .659 | -.382 | 1 | .656 | -.656 | -.180 | -.049 | -.025 |
|  | Sig. (2-tailed) | .172 | .002 | .001 | .079 |  | .001 | .001 | .423 | .830 | .913 |
| LFnu | Pearson Corr. | -.247 | -.819 | .907 | -.544 | .656 | 1 | -1.000 | -.120 | .333 | -.123 |
|  | Sig. (2-tailed) | .267 | .000 | .000 | .009 | .001 |  | 0.000 | .593 | .129 | .587 |
| HFnu | Pearson Corr. | .247 | .819 | -.907 | .544 | -.656 | -1.000 | 1 | .120 | -.333 | .123 |
|  | Sig. (2-tailed) | .267 | .000 | .000 | .009 | .001 | 0.000 |  | .593 | .129 | .587 |
| Total  power | Pearson Corr. | .248 | .001 | -.018 | .787 | -.180 | -.120 | .120 | 1 | .456 | -.101 |
|  | Sig. (2-tailed) | .265 | .996 | .937 | .000 | .423 | .593 | .593 |  | .033 | .656 |
| VLf | Pearson Corr. | -.128 | -.360 | .393 | .158 | -.049 | .333 | -.333 | .456 | 1 | -.389 |
|  | Sig. (2-tailed) | .569 | .100 | .070 | .482 | .830 | .129 | .129 | .033 |  | .073 |
| IAP | Pearson Corr. | .451 | .212 | -.276 | .030 | -.025 | -.123 | .123 | -.101 | -.389 | 1 |
|  | Sig. (2-tailed) | .035 | .344 | .214 | .894 | .913 | .587 | .587 | .656 | .073 |  |

Note: S_Entropy: sample entropy; Higuchi: Higuchi fractal dimension; MFDFA: modified detrended fluctuation analysis, RMSSD: root mean square of successive differences between normal heartbeats; VLF: very low frequency spectrum; LF nu: indexed low frequency spectrum, HF nu: indexed high frequency spectrum; LF/HF: low frequency high frequency ratio
