# Supplementary material for: External low energy electromagnetic fields affect heart dynamics: surrogate for system synchronization, chaos control and cancer patient’s health
Source: Front Netw Physiol. 2025 Jan 3;4:1525135. doi: 10.3389/fnetp.2024.1525135 (PMC11739291; doi:10.3389/fnetp.2024.1525135)
Supplement: Supplementary file 2 [file DataSheet1.pdf]

Supplement information

Figure 1S. Representation of patient's dynamic behavior with the application of different logistic equation.

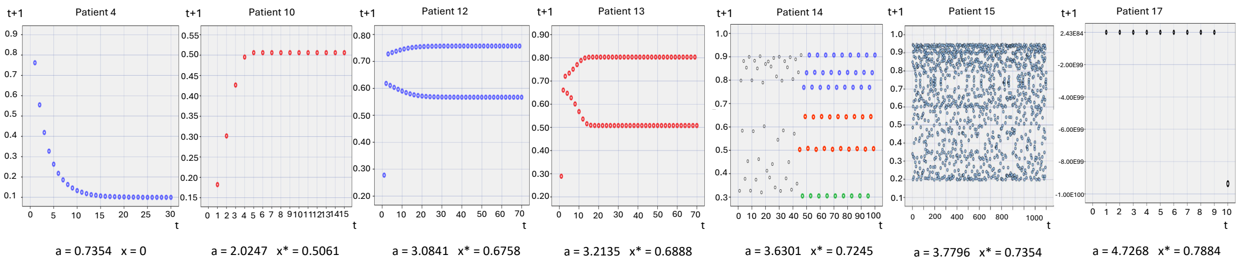

Constant a	Patient's dynamics
ß 1.0000	Orbits attracted to the trivial solution x = 0
Between 1 and 3	The fixed point is stable and attracts all trajectories
3.000	The fixed point becomes unstable. Start of the chaotic region
3.570	Accumulation point of cycles with periods 2^n
3.678	The first odd-period cycle appears
3.284	The period-three cycle appears
4.000	End of the chaotic region
> 4.000	The fixed point tends towards infinity

Table 1S. Results from logistic difference equation for 22 cancer patients at their first exposure to EMF.

Magnitude	Patient's initial	Patient_ID	OS (Days)	Status	Constant a	Fixed point	Slope
0 < a < 1	LS	1	654	1	0.0167	There is no in the 0 < x < 1 interval	1.9833
	MA	2	199	1	0.2461		1.7539
	RS	3	285	1	0.4481		1.5519
	JP	4	119	1	0.7354		1.2646
	MAu	5	72	1	0.8387		1.1613
1 < a < 3	VR	6	85	1	1.0601	0.0567	0.9399
	PJF	7	303	1	1.1871	0.1576	0.8129
	EB	8	505	0	1.4203	0.2959	0.5797
	JAS	9	861	1	1.4856	0.3269	0.5144
	SS	10	353	1	2.0247	0.5061	-0.0247
	VLG	11	359	1	2.1557	0.5361	-0.1557

3 < a < 4	CMR	12	574	1	3.0841	0.6758	-1.0841
	NJP	13	946	1	3.2135	0.6888	-1.2135
	MS	14	318	1	3.6301	0.7245	-1.6301
	HMC	15	559	1	3.7796	0.7354	-1.7796
a > 4	MACT	16	480	1	4.1471	0.7589	-2.1471
	KSV	17	876	1	4.7268	0.7884	-2.7268
	JBC	18	1178	0	5.5348	0.8193	-3.5348
	MNO	19	1019	1	6.5053	0.8463	-4.5053
	PA	20	515	1	6.6076	0.8487	-4.6076
	JCC	21	240	1	6.6778	0.8503	-4.6778
	LG	22	562	0	7.1311	0.8598	-5.1311

Note: OS: overall survival; status 0: alive 1: dead; fixed point defined by $x^* = 1 - 1/a$; inclination defined by $l = 2 - a$.

Table 2S. Pearson Correlation between different HRV metrics and constant a for 22 cancer patients at their first exposure to EMF.

		SEntropy RRI	Higuchi RRI	mfdfa group_RRI	RMSSD RRI	LF/HF	LFnu	HFnu	Total power	VLf	IAP
SEntropy RRI	Pearson Corr.	1	.528	-.363	.278	-.302	-.247	.247	.248	-.128	.451
	Sig. (2-tailed)		.012	.097	.211	.172	.267	.267	.265	.569	.035
Higuchi RRI	Pearson Corr.	.528	1	-.912	.440	-.613	-.819	.819	.001	-.360	.212
	Sig. (2-tailed)	.012		.000	.041	.002	.000	.000	.996	.100	.344
MFDFA group_RRI	Pearson Corr.	-.363	-.912	1	-.477	.659	.907	-.907	-.018	.393	-.276
	Sig. (2-tailed)	.097	.000		.025	.001	.000	.000	.937	.070	.214
RMSSD RRI	Pearson Corr.	.278	.440	-.477	1	-.382	-.544	.544	.787	.158	.030
	Sig. (2-tailed)	.211	.041	.025		.079	.009	.009	.000	.482	.894
LF/HF	Pearson Corr.	-.302	-.613	.659	-.382	1	.656	-.656	-.180	-.049	-.025

	Sig. (2-tailed)	.172	.002	.001	.079		.001	.001	.423	.830	.913
LFnu	Pearson Corr.	-.247	-.819	.907	-.544	.656	1	-1.000	-.120	.333	-.123
	Sig. (2-tailed)	.267	.000	.000	.009	.001		0.000	.593	.129	.587
HFnu	Pearson Corr.	.247	.819	-.907	.544	-.656	-1.000	1	.120	-.333	.123
	Sig. (2-tailed)	.267	.000	.000	.009	.001	0.000		.593	.129	.587
Total power	Pearson Corr.	.248	.001	-.018	.787	-.180	-.120	.120	1	.456	-.101
	Sig. (2-tailed)	.265	.996	.937	.000	.423	.593	.593		.033	.656
VLf	Pearson Corr.	-.128	-.360	.393	.158	-.049	.333	-.333	.456	1	-.389
	Sig. (2-tailed)	.569	.100	.070	.482	.830	.129	.129	.033		.073
IAP	Pearson Corr.	.451	.212	-.276	.030	-.025	-.123	.123	-.101	-.389	1
	Sig. (2-tailed)	.035	.344	.214	.894	.913	.587	.587	.656	.073	

Note: S_Entropy: sample entropy; Higuchi: Higuchi fractal dimension; MFDFA: modified detrended fluctuation analysis, RMSSD: root mean square of successive differences between normal heartbeats; VLF: very low frequency spectrum; LF nu: indexed low frequency spectrum, HF nu: indexed high frequency spectrum; LF/HF: low frequency high frequency ratio
